# Reporting and valuing one’s own health: a think aloud study using EQ-5D-5L, EQ VAS and a time trade-off question among patients with a chronic condition

**DOI:** 10.1186/s12955-020-01641-4

**Published:** 2020-12-17

**Authors:** Olivia Ernstsson, Kristina Burström, Emelie Heintz, Helle Mølsted Alvesson

**Affiliations:** 1 QRC Research Unit, Department of Learning, Informatics, Management and Ethics (LIME), Karolinska Institutet, Tomtebodavägen 18A, 171 77 Stockholm, Sweden; 2 Health Outcomes and Economic Evaluation Research Group, Stockholm Centre for Healthcare Ethics, Department of Learning, Informatics, Management and Ethics, Karolinska Institutet, 171 77 Stockholm, Sweden; 3 Health Care Services, Region Stockholm, 171 77 Stockholm, Sweden; 4 Health Systems and Policy, Department of Global Public Health, Karolinska Institutet, 171 77 Stockholm, Sweden

**Keywords:** EQ-5D-5L, EQ VAS, Patient-reported outcome measures, Qualitative interviews, Think aloud, Time trade-off

## Abstract

**Background:**

The EQ-5D-5L, the EQ VAS, and the time trade-off (TTO) are commonly used to report and value health. Still, there is a need to better understand how these questionnaires and methods are perceived by the respondents, as well as the thoughts and motives behind their responses. The aim of this study was to increase knowledge of how individuals think and reason when reporting and valuing their own current health, using EQ-5D-5L, EQ VAS, and an open-ended TTO question.

**Methods:**

Twenty patients with type 1 diabetes participated in qualitative individual think aloud interviews in Stockholm, Sweden. Participants were asked to describe their thoughts when responding to three assessments. The interviews were transcribed verbatim and analyzed using thematic analysis.

**Results:**

The analysis showed that participants conducted the assessments by contextualizing and interpreting instructions, relating the questions to their own health, using different recall periods and time perspectives, and using personal, interpersonal, or normative comparators. It was challenging to reduce the experience of everyday life into a response option, and the thoughts behind the responses differed between the assessments. Before deciding on what to include, participants thought of the purpose and context of the assessments. Current health or past experiences of health were applied in the EQ-5D descriptive system and in EQ VAS, while participants focused on the future in the TTO. Thoughts about the impact on others, personal goals, and expectations on future health were more clearly integrated in the TTO assessment. All participants considered the trade-off between life years and health. However, despite the use of different comparators, the concept of ‘full health’ was found difficult to imagine or relate to.

**Conclusions:**

This study provides insights as to how responses to the EQ-5D-5L, EQ VAS, and TTO assessments are complementary and where these assessments differ in adults with a chronic condition. The findings may contribute to a better understanding when interpreting the quantitative results and contribute to the literature pertaining to possible explanations for differences in health state values depending on the valuation method.

## Introduction

Patient-reported outcome measures (PROMs) provide standardized measures of health status or health-related quality of life from a person’s own perspective [1]. These measures are used to describe and monitor health, to inform patient management, and to assess the quality and effectiveness of health care [1–3]. A commonly used generic PROM is the EQ-5D, which enables health state classification by means of a descriptive system and provides an indirect method for health state valuation [4]. The EQ-5D health state values have been elicited through valuation methods such as the time trade-off (TTO), or the visual analogue scale (VAS) [3]. Although these questionnaires and methods are commonly applied in clinical settings and in health economic evaluation, there is still a need to better understand how they are perceived by the respondents, as well as the thoughts and motives behind their responses.

In the TTO valuation method [5], respondents are asked to choose between living a certain period of time (e.g., 10 years) in a health state less than full health, or to trade off years to live a shorter time in full health. Although TTO is often referred to as *one* valuation method, values can differ as a result of using different mode of administration, elicitation procedure, phrasing, visual aids, time frame, and smallest tradeable unit [6, 7]. Other considerations include whether health state valuations should be conducted by individuals in the general population or in a specific patient population, and if the respondents should value their own current health state or a hypothetical health state [8–12].

Previous qualitative studies exploring responses to EQ-5D have mainly focused on how respondents perceive the questionnaire. The feasibility, validity, acceptability, and perceived relevance of EQ-5D have been examined with varying results in different patient groups [13–17]. Other studies have analyzed answers to open-ended questions after standardized interviews or surveys to better understand how the questionnaires are perceived or approached by the respondents. For example, previous studies suggest that there are several important health aspects in addition to those covered by the EQ-5D descriptive system [18], and that VAS valuations of hypothetical health states may be too complex for self-completion [19].

Moreover, previous qualitative research on health state valuations have mainly explored how respondents value hypothetical health states [20–23]. Think aloud studies in the TTO method have shown that respondents take into account both their past experiences and their imagination, and consider what impact the hypothetical health states may have on their own lifestyle as well as on others [20–22]. Other study findings suggest that respondents find certain hypothetical health states difficult to imagine [20–22], and consider the impact on self and others as well as the duration of a health state to a larger extent in the TTO method, compared to the VAS method [23]. However, none of the previous identified studies have examined thought processes when using the TTO method to value respondents’ own current health.

The need to further explore cognitive processes behind responses to PROMs and valuation of health states has been emphasized, i.e. to explore how and why individuals answer in the way that they do [9, 19, 24]. Thus, there is a need to better understand how individuals report and value their own health, preferably by exploring thought processes of patients with personal experiences of health problems. The aim of the present study was to increase knowledge of how individuals think and reason when reporting and valuing their own current health using the EQ-5D-5L descriptive system, EQ VAS, and an open-ended TTO question. For this study, qualitative interviews were conducted with patients with type 1 diabetes.

## Methods

### Setting

The study was conducted in Stockholm, Sweden. In Sweden, health care is primarily financed through taxes, and there is a national benefits system which limits maximum annual costs for health care for an individual [25]. The 21 regions have the responsibility of providing primary and specialist health care. Region Stockholm is the largest with approximately 2.3 million residents [25, 26].

Study participants were recruited from the academic specialist clinic Center for Diabetes. Adult patients with type 1 diabetes are referred to the clinic and attend health care visits approximately once a year. Care is provided by teams of physicians, diabetes specialist nurses, dietitians, physical therapists, and podiatrists.

### Study design and participants

This was a qualitative interview study, and the methods are reported in accordance with the Consolidated criteria for reporting qualitative research (COREQ) [27]. The purposeful sampling [28] included patients with type 1 diabetes as it is a chronic disease that impact the daily life and may affect several dimensions of health. It was expected that those who report less than full health would be more prone to consider the trade-off between life years and health status compared to individuals not experiencing any health problems. The inclusion criteria were men and women between 18‒70 years of age who have had type 1 diabetes for at least 5 years. For feasibility reasons only persons speaking Swedish well enough to participate in an interview, as assessed by the recruiting nurses, were included.

**Fig. 1:**
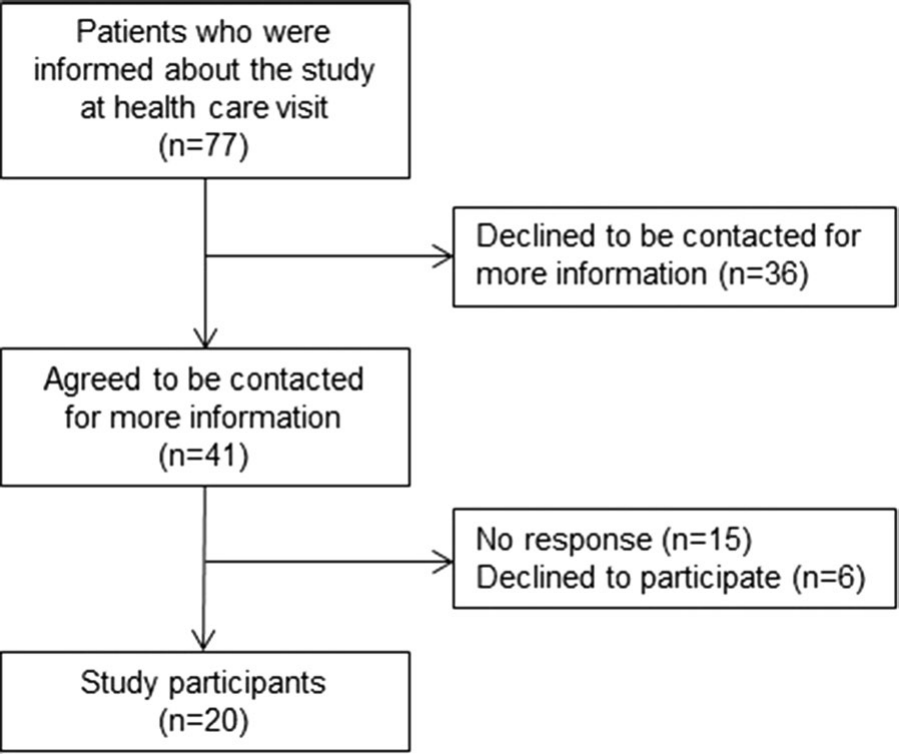
Description of recruitment of interview participants

Four nurses provided written and verbal information about the study to patients who met the inclusion criteria during regular health care visits at Center for Diabetes. The specific weeks for recruitment (i.e., one week each in January, March, and June, 2018) were selected based on the schedule of the clinic. Patients who provided their telephone number during the initial phase of recruitment were contacted by the first author. Twenty out of the 77 patients who received information about the study participated (Fig. 1).

### Data collection and analysis

The interviews were guided by think aloud technique and a semi-structured interview guide [29]. Prior to the main data collection, three pilot interviews were conducted after which minor adjustments were made to the interview guide (i.e., wording and length). The final interview guide consisted of questions regarding the experience of the onset of symptoms and the diagnosis, and experience of other diseases, followed by the think aloud tasks and probing questions for EQ-5D-5L, EQ VAS, and TTO, respectively (described below). Finally, participants could add or elaborate on their thoughts at the end of the interview. Afterwards, brief background characteristics of the participants were collected through a list of structured question (Table 1). Apart from the self-reported health assessments, no other information regarding the participants’ medical conditions was collected. However, 13 participants described during the interviews that they also experienced other health problems (e.g., asthma, cardiovascular disease).

**Table 1:** Participants’ background characteristics

	Participants, n
Men	13
Women	7
Age	
< 30	3
30–39	5
40–49	5
50–59	1
≥ 60	6
Highest educational level	
Elementary school (9 years)	0
Upper secondary school (11–13 years)	6
University < 3 years	5
University 3 years or more	9
Years since diagnosis	
5–9 years	1
10–19 years	3
20–29 years	7
30–39 years	7
40–49 years	0
≥ 50 years	2
Living together with someone	17
Having children	13

The three assessments included were the EQ-5D-5L descriptive system, EQ VAS and TTO. The EQ-5D-5L descriptive system covers five health dimensions (mobility, self-care, usual activities, pain/discomfort, and anxiety/depression), each with five severity levels (no, slight, moderate, severe, and extreme problems) [30]. The EQ VAS is a vertical scale with the end points 100 (the best health you can imagine) and 0 (the worst health you can imagine). An open-ended TTO question was used for the participants’ valuation of their current health. The TTO question consisted of a horizontal line representing 0–10 years (every year was marked and labelled 0, 1, 2…, 10 years and every half year was marked, but not labelled) and read: ‘Imagine that you are told that you have 10 years left to live. In connection with this you are also told that you can choose to live these years in your current health state or that you can choose to give up some life years to live for a shorter time period in full health.’ The respondent was asked to indicate the number of years in full health that would be of equal value to 10 years in his or her current health state on the scale provided. Below the line, there was an additional sentence in parenthesis: ‘if you think that you currently have full health, you should mark 10 years’. This open-ended TTO question has been used in the development of experience-based value sets for EQ-5D-3L [31] and EQ-5D-5L [32], and similar versions have been included in other studies [33–35].

Participants were handed paper versions for each of the three assessments and were asked to describe everything they thought of while responding. Individual responses to the three assessments (i.e. EQ-5D-5L descriptive system, EQ VAS, TTO) are presented in Table 2.

**Table 2:** Participant characteristics and health profiles on the EQ-5D-5L descriptive system, EQ VAS score and reported number of years in full health on the time trade-off question

Participant	Sex	EQ-5D-5L profile	EQ VAS score	TTO response (years in full health)
P1	Male	11111	70	10
P2	Male	11111	85	10
P3	Female	11111	75	10
P4	Female	11112	88	9
P5	Male	11111	85	10
P6	Male	11111	85	0 (10)^a^
P7	Male	41432	75	10
P8	Male	11121	80	10
P9	Male	11121	70	10
P10	Female	11111	80–85	10
P11	Male	11121	85	9.5
P12	Male	11111	60–70	–
P13	Female	31221^c^	55	–
P14	Male	11111	60	10
P15	Female	11111	95	10
P16	Female	11221	95	10
P17	Male	11112	95/81^b^	10
P18	Male	11112	70	10
P19	Female	41233	30	10
P20	Male	31132^d^	70	–

EQ-5D-5L profile describes the severity levels on each of the five health dimension, i.e., numbers representing no (level 1), slight (level 2), moderate (level 3), severe (level 4), and extreme problems (level 5)

^a^Did not want to “give up any years” (i.e., 10 years in full health), but answered 0 on paper

^b^Responded 95 according to own reference point, and 81 in comparison with others

^c^Changed from the initial response 31,231 during interview

^d^Changed from the initial response 11,132 during interview

The data collection was completed after 20 individual interviews (28–90 min) that took place either in a non-medical room at a conference center adjacent to the Center for Diabetes or at Karolinska Institutet, between February and July, 2018. The sample size was based on reflections on sufficient information power, which depends on study aim, sample specificity, use of established theory, quality of dialogue, and analysis strategy [36]. From the 14th interview and onwards, the first and last author discussed to what extent the additional interviews provided new or contradictory findings. All interviews were performed by the first author (MSc), who was working as a PhD student in health economics and outcomes research at the time of the study. The interviewer and the participants had no established relationship prior to the study.

**Fig. 2:**
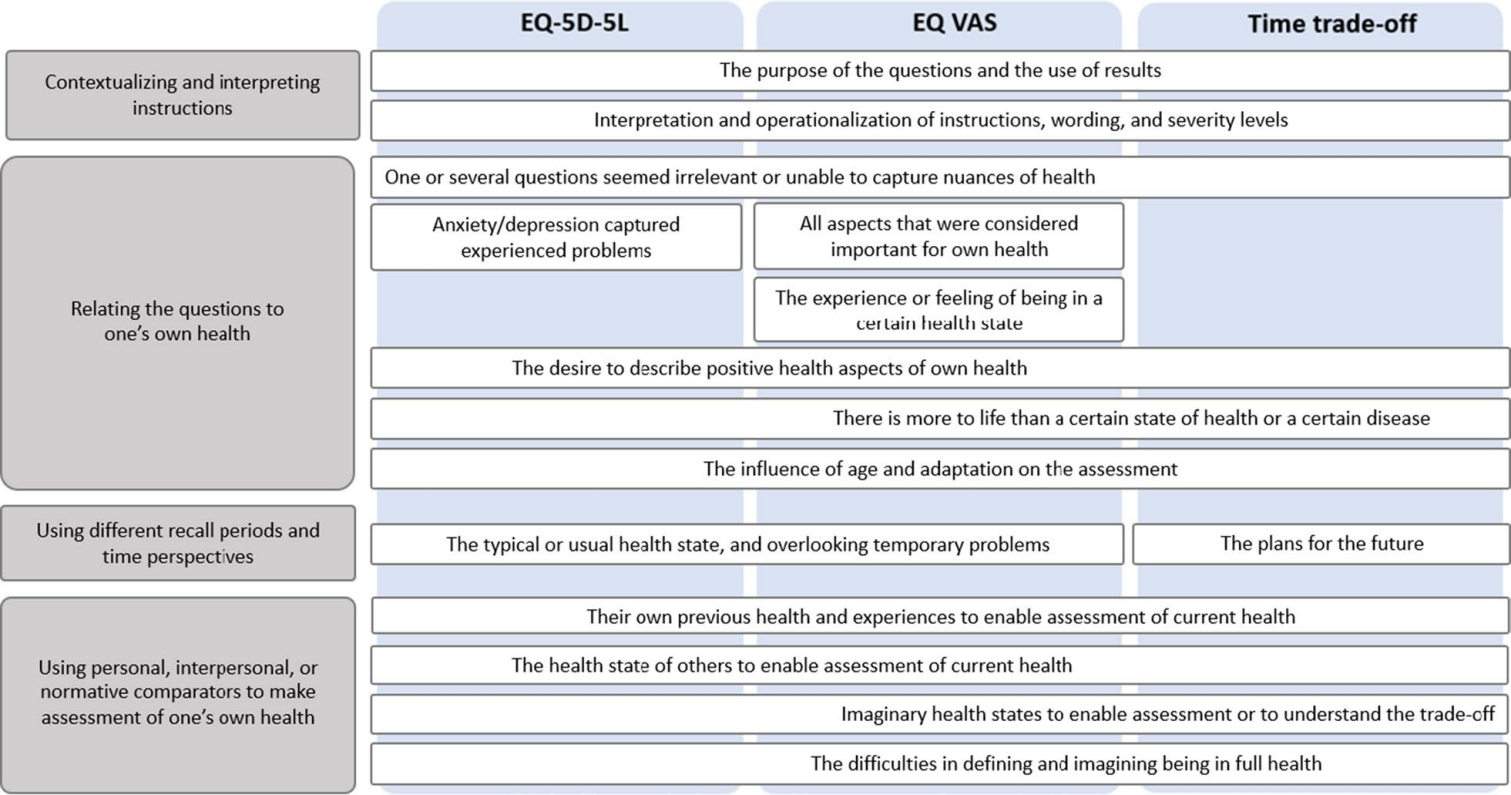
Overview of the analysis of participants’ thoughts when reporting and valuing their own health, using the EQ-5D-5L descriptive system, EQ VAS, and an open-ended TTO questions. *Note*: Some codes mainly address one or two of the assessments, which is indicated by the location of the text in the figure

All interviews were audio recorded, transcribed verbatim in Swedish, and managed and coded in NVivo 12 software. The data was analyzed using a qualitative thematic analysis [37]. To begin with, the first author read all the transcripts and conducted initial coding of each interview separately. For the first two interviews, all the authors read the transcripts and discussed the initial coding. Second, the first author reviewed the codes in all the transcripts by re-reading the findings addressing EQ-5D-5L, EQ VAS, and TTO separately. The analysis was an iterative process that advanced from description of patterns in the data (i.e., codes) to interpretation of meaning for the entire data material (i.e., categories and themes) (Table 3). The codes that addressed the three different assessments were assembled before categories and themes were developed. Nevertheless, some findings mainly addressed one or two of the assessments (see Fig. 2). The categorization and interpretation were discussed and reconciled between the authors. The quotes presented in the results section were translated from Swedish.

**Table 3:** Example of how a statement was coded and informed the development of categories

Statement	Code	Category
*The first thing I thought of was the physical health. And then I thought of what I- where I was physically, in terms of my physical performance last year at about the same time.. And then I would say it’s a little worse now when it comes to my endurance (..). But I am stronger than I was then*	Participants thought of: their own previous health and experiences to enable assessment of current health	Using personal, interpersonal, or normative comparators to make assessment of one’s own health

### Ethical considerations

The study was approved by the Regional Ethical Review Board, Stockholm (dnr 2017/526-31, 2017/2123-32). Written informed consent was obtained prior to the interviews. Each participant received two cinema tickets as compensation for the time spent on the interview. After each interview, participants were given contact information of a counsellor at the Center for Diabetes. The nurses who initiated the recruitment were not informed about who participated in the study.

## Results

The analysis of how participants think and reason when reporting and valuing their own health resulted in two themes that were informed by four categories (Fig. 2).

### Theme 1: Personalizing questions and considering what aspects to include in the response

#### Contextualizing and interpreting instructions

Participants expressed thoughts about the purpose of the questions and the use of results when reasoning about what to include in the assessment. The reflections concerned in what situations and for what purpose the assessments were being administered, by whom the questions were asked, how answers could be interpreted in a meaningful way, and whether their answers could lead to any action.

I can feel like (…). Should I answer in general or should I answer for a certain period or, what do you want? /laughs/ Is it the last few weeks or the last few months or is it a certain time period I should state – or is it over the year or in general (…). I can’t just – like I could just look at today and I can easily answer, but you don’t really know what- what does a doctor or the person who receives this- what do they really want to know, is it just how I am feeling right now or how I have been lately? (Participant 5)

Another issue that was raised was whether certain aspects (such as those related to diabetes) or general health status should be considered. As participants struggled to make judgements of what aspects to include in their assessments, they expressed a desire to explain the response and to discuss potential areas for health improvement.

It is really difficult (…). Anxiety and depression, it is such a broad concept. You can be very anxious about your blood sugar, it just goes crisscross in different directions (…). But I perceive it more like, yes you are feeling down and it is about bigger stuff than… Well, you would need “are you feeling down because of your blood sugar level”, or that you clarify (…). **Why** are you anxious or depressed – that’s it, you need those follow-up questions. (Participant 16)

The questions were approached through the participants’ interpretation and operationalization of instructions, wording, and severity levels. The instructions for the EQ-5D-5L descriptive system and EQ VAS were generally comprehensible. At the same time, it was challenging to reduce the experience of one’s own health and everyday life into one response option or into a number. For example, it was considered difficult to describe the severity of problems when these were severe or extreme in certain situations but rarely occurred.

The interpretation of questions varied, which was especially clear in the anxiety/depression dimension. Interpretations varied regarding the perceived severity (e.g. being diagnosed with anxiety or depression or experiencing worry or sadness as a natural part of life). Furthermore, some interpreted the question strictly as worrying about their personal health condition, while others also considered worrying about someone close. The dimension was generally interpreted as two separate constructs (anxiety and depression), which was not found to be the case in the dimension for pain/discomfort. In this case, participants did not report problems on the EQ-5D-5L descriptive system although they experienced discomfort in their everyday life. In this question, discomfort was interpreted as a consequence of pain.

What is discomfort? I have, after all, I have no pain. But I have problems with low blood sugar (..) I just saw ‘pain’ and thought well I have no pain! But I can say, on the other hand, that I have quite severe problems with the blood sugar level and how much that affects my life. But I would never interpret… I think of the musculoskeletal system. Muscle pain or pain in the joints, something like that. So no thought of diabetes at all. (Participant 3)

In the TTO valuation, all participants considered the trade-off between life years and health. At the same time, some participants expressed the need to read the instructions several times. All but two participants provided a response to the TTO question. The two participants who did not respond to the question expressed explanations that showed that they understood the instructions for the TTO question. When challenges regarding the TTO were raised, some expressions included that it was perceived as difficult or complex to imagine and respond to (e.g. to simultaneously consider current health, full health, and number of years) or that it represented ideas about the future that were too hypothetical.

What kind of question is this? I can’t predict the future or fantasize about it either. Here you put two things against each other. So, of course I want full health, and for as long as possible. And since I don’t know what it’s like to be in full health, how am I supposed to answer that? (Participant 20)

Participants asked follow-up questions when the TTO question was not fully understood, due, for example, to difficulties understanding that the scenario concerned their own current health rather than future health deterioration, or where to put the mark on the line from zero to ten years.

#### Relating the questions to one’s own health

Participants conducted the assessments by relating the questions to their own health. Some participants thought one or more dimensions in the EQ-5D-5L descriptive system were not relevant, or that it required effort to link the dimensions and severity levels to the experiences from everyday life. Some dimensions seemed to be intended for someone older or someone with a more severe condition, which was expressed especially by participants who felt that they lived a healthy life or had learnt how to manage their health problems. Other aspects that were considered important for the participants particular health situation included, for example, sleep, stress, and physical capabilities (i.e. not merely the absence of problems). Furthermore, participants expressed that they experienced a burden from different treatments, which was not captured by the descriptive system. On the other hand, the anxiety/depression dimension was described as relevant in capturing worries about future risks of diabetes-related complications.

But you also get a bit worried if you start thinking about your diabetes… Because you know all the time that you need to have a constant very good blood sugar level or it will affect feet and eyes and those things. (Participant 18)

In addition to reporting health problems, participants described a desire to report capabilities or aspects that were associated with health rather than disease. For example, instead of reporting no problems with mobility or anxiety/depression, there was a desire to report abilities (such as being physically strong or feeling happy). Areas in which the participants wanted to experience improvements were identified, also by those who did not report any problems in the five dimensions. Some concerns were raised regarding the fact that health questionnaires are often too problem-oriented and too focused on what is not desirable. This concern was noticed among participants who found the health dimensions relevant and among those who did not, as well as among those who reported problems and among those who did not.

And then I think, mobility is also way too blunt in one direction (…). I would love to have something in the other direction as well. Like do you work out actively or is it just that you can walk here or is it that you run (…). When I see this I think about old ladies with heart failure. Sorry, no but like this is just – no I don’t have problems in walking about. Okay. But if I actually run the half marathon, where is that in this scale? (Participant 3)

The EQ VAS enabled participants’ individual interpretations of what aspects to include in the assessment of their current health. Participants expressed that they could include all aspects that were considered important to their health status. The response comprised an overall health assessment, which could include diabetes and other health-related issues as well as satisfaction with their lifestyle, leisure time, job, and relationships. Health aspects that were felt not to be covered by the descriptive system were included, such as physical activity, diet, sleep, and stress. Furthermore, the EQ VAS assessment was described as representing their experience or feeling about being in a certain state of health.

I think it is hard for someone else to understand, really… If you have the facts, my diseases and so on… And then see this number. It is probably quite difficult to understand. I can feel so damn healthy and I am actually so sick, right. (Participant 6)

Participants expressed the opinion that there is more to life than a certain health state or a disease. Foremost, the health assessment became complicated when participants on the one hand acknowledged having some health problems, but on the other hand did not consider them to be obstacles. The perceived differences between having diabetes, having problems, and considering them to be obstacles were seen in all three assessments. This finding was especially clear in the TTO assessment, in which only problems that prevented participants from doing things they wanted to do were considered in the trade-off.

You don’t value your physical health in that sense, but you value the emotional health here, of having accomplished something, so (…). You don’t necessarily measure how you feel, but you measure… How you perceive that your problems are holding you back from things you would like to do, in a sense. (Participant 17)

The reasoning around what to include when responding activated thoughts about self-image and thoughts of living with a chronic disease (i.e. having, in contrast to being). Some participants reflected on how their own adaptation in terms of both expectations and activities might have influenced their responses. Examples included having become used to the experience of health problems or having adapted their activities (e.g. biking instead of walking).

I like to be correct /laughs/ (…). There are very big concepts in these questions and it has to be that way. But, like this, yes I can do everything I do, because I do it in a different way than what I might normally do. So, then it will be like, what should I really answer – I can do everything, but I feel pain. (Participant 4)

Likewise, some reasoned around the idea that a person’s age is likely to influence the responses on all three assessments, as a consequence of shifting perspectives, lifestyle, and expectations on one’s health when aging.

[EQ VAS] What do I think of when I say 70… I think I’m getting old (..) I’ve had diabetes for quite a long time. /Sighs/ And that you know that the diseases come when you get older, and there have been some… and diabetics are damn well vulnerable to most things. I think (..) if you can maintain your health for a while, but, I would like to get up to 90 at least, or 80, that’s my idea. (Participant 20)

A common explanation for not wanting to trade any life years in the TTO assessment was that participants considered their lives to be good and they experienced no or limited problems. Even when experiencing problems, participants expressed not wanting to give up other parts of life. Thus, also aspects that were not directly health-related were included in the TTO. Some motives included wanting to spend as much time as possible with family and to see their children grow up. Furthermore, the question did not only concern the participants’ own situation, as it was also considered important to give as much time as possible to close family or those depending on them. Some participants explained that their decision was based on prioritizing the needs of others over their own*.*

If I would decide to… become, well, entirely healthy according to that and live in full health, which would mean that I would have my sight and not have diabetes or anything, and get to choose (…). Now you didn’t say exactly how many years to give up but it doesn’t really matter – but we say that – yes if you live five years instead of ten years. Then it… It would be very – for me, it would be very egoistic. To do it for my own sake, when I still have children and grandchildren. That’s my spontaneous thinking. (Participant 7)

### Theme 2: Using reference points and comparators to enable assessment of one’s own health

#### Using different recall periods and time perspective for the assessments

There were variations in whether participants considered their health at that present time or over a longer period of time (e.g. the last weeks or months) when responding to the EQ-5D-5L descriptive system and EQ VAS. An explanation for applying a longer time perspective was that a health state is considered to be relatively constant over time. Although the instruction to consider “your health today” was noticed, responses to the EQ-5D-5L descriptive system were based on the typical or usual health state while temporary health problems were mostly overlooked. Temporary problems were not part of how they perceived their overall health, or they knew the cause of the problem and considered it to be irrelevant for the assessment (e.g. pain as a consequence of yesterday’s exercise).

[EQ VAS] It says today, but I think it is more maybe the last week or last month or months, even, that it is being included somehow. But I think maybe it is more about my definition of health. Just because my definition is physical activity and diet. So it doesn’t matter that much what I did today. It is more about what I have done the last weeks, months – that is what is important in how I feel today. So it probably has to do with my definition of health rather than the fact that I just ignored that is says today. (Participant 10)

In contrast, the focus clearly shifted from considering past and current health status to considering the future and planning ahead when responding to the TTO question.

The last one, with the years… That is more about my view of the future. Or yeah – in my opinion, it is more about how I view the future than how I am actually feeling. (Participant 10)

Participants considered their plans for the future, expectations, and the things they would like to do or accomplish before they die. Some expressed having less focus on their current health status as it was self-evident and part of normality, thus they shifted their focus to their bucket list.

No I thought more of… what I have left to do /says laughing/. So, I – like what you have on your little bucket list, that you want to do, yeah then I have to start doing these things. (Participant 9)

Furthermore, the responses to the TTO question were influenced by expectations regarding future health status and longevity. Ten years with their current health was already considered to be a loss by participants who expected to live longer. In contrast, when expecting health deteriorations that would affect quality of life in the coming years, ten years in current health was considered a gain. Although thoughts about the risk of future diabetes complications were present in the EQ-5D-5L and EQ VAS assessments, it was even more clearly considered in the TTO assessment.

#### Using personal, interpersonal, or normative comparators to make assessment of one’s own health

Different reference points and comparators were used to facilitate the assessments. In the search for reference points, participants reasoned around the subjective nature of the health assessments. Consequently, their usefulness was questioned by some. In all three assessments, participants made comparisons with their own previous health status or previous experiences (e.g. by comparing with a situation one year ago, or when being very ill), and comparisons were less commonly made with the health status of others.

If you should limit it to today, then you have to have like a parameter that you can… relate it to. That this was, this was how much worse it was back then. (Participant 16)

In the EQ VAS assessment, participants either considered best imaginable health as an external reference (i.e. other people, or an imagined ideal state) or considered what would be possible to achieve based on their own circumstances. Less attention was given to the interpretation and comparison to worst imaginable health. Some participants focused on specific parts of the scale for the positioning of their current health, for example by considering the scale between 50 and 100. Furthermore, participants made their own judgements regarding what numbers were considered satisfactory or good.

But when I look at this scale, I think that one hundred is a professional athlete, and… just getting by in everyday life is fifty (…) And there in between, I should find where I’m at. And since I feel that I am a little bit behind right now, I’m thinking that I can’t be that high above fifty. (Participant 14)

Most participants related their health to 100 (best imaginable health) in the EQ VAS assessment. For example, the responses were explained by describing what it would take to be able to answer 100. The areas for improvement were focused on aspects that were considered as their own responsibility (e.g. exercise, diet, and preventing future complications).

There might be others who have one hundred percent, I don’t know /laughs/ (…) Should one sit here and say that things can’t get better? (Participant 5)

The concepts of the best health you can imagine and full health were central to the application of normative comparators (e.g. comparing one’s health to an ideal). These concepts were found difficult to define, to imagine, or to relate to.

It… it could be like this. Eh. What is the definition of full health here? It comes back to… My own reference or a global reference. Because if you say full health that you think is equivalent to live ten years in your current health, then I would say that your own reference may not be worth as much, even if you have full own reference health. I mean full health for me now, as I said, sure when I was 20 (…) With 100 percent using my own frame of reference, I might still not have been able to run a marathon if it had been what I wanted. Should I consider full health from sort of global perspective, like, the question is interesting, but I think one must define full health somehow so that you really get a comparable reference. (Participant 17)

In EQ VAS, although participants focused on areas for improvements, some expressed that the goal was not to achieve the best imaginable health. The current situation was described as manageable and the problems experienced in everyday life as “normality”. The distinction between one´s own health and full health became even more complicated among those who expressed that having diabetes has led to a healthier lifestyle and a positive impact on their overall health status.

Participants expressed difficulty imagining being in full health in the TTO assessment. Some explanations included not having any personal experience of being in full health or not expecting to be in full health in the future.

You never get that opportunity… To live in full health, that’s not an option that exists in reality. Like, what you can do is to live as good as you can with the time you have left, I think… (Participant 18)

The two participants who did not provide an answer to the TTO assessment explained that the question was impossible since full health was not a realistic scenario.

This wasn’t easy… /sighs/ because my current health state – I will never get full health – I am diabetic – it is a chronic disease! And I have been my entire life! So I don’t know what it would be like to be in full health… No I – I can’t answer it. Sorry! (Participant 13)

Finally, participants considered imaginary health states in order to understand what it would take to give up life years in the TTO. Examples were mostly concerned with future deterioration in health and the risk of diabetes complications, and participants used their imagination as well as their vicarious experiences*.*

Well if I had kidney failure and other things, the answer might have been different. But as it is now, this is it. (Participant 7)

## Discussion

Respondents’ interpretation of standardized instruments is challenging to capture, and this study was conducted to better understand the thought processes when respondents report and value their current health. Our findings showed that respondents conduct the assessments by contextualizing and interpreting instructions, relating the questions to one’s own health, using different recall periods and time perspectives, and using personal, interpersonal, or normative comparators.

Our findings indicate that respondents consider a wide range of health aspects and well-being when assessing their own health status, which is in line with previous studies based on survey responses regarding hypothetical health states [18, 19]. For example, some participants expressed the desire to describe “positive health aspects” rather than the degree of problems. The desire to report both problems experienced and abilities can be related to the well-known definition of health as a “state of complete physical, mental and social well-being and not merely the absence of disease or infirmity” [38]. Yet, some participants expressed a contradiction between having a chronic disease and at the same time feeling healthy.

It is important to consider the possible influence of response shift and adaptation when interpreting results from PROMs and valuations of experienced health states. Previous research has suggested that changes in internal standards, values, or conceptualization of health can occur over time [39, 40], and having a chronic disease is likely to have influenced the thought processes in all three assessments. In a previous study on experience-based TTO valuations among individuals with diabetic retinopathy, adaptation was mentioned as a possible explanation for the relatively higher TTO values for moderate visual impairment compared to no or mild visual impairment [41]. Similarly, participants in our study expressed thoughts about adaptation and expectations of the future during the TTO valuations.

For all three assessments, we found that participants expressed thoughts in line with established theoretical models of cognitive processes, including comprehension, retrieval/sampling strategy, standard of comparison, and judgement, before reporting and selecting a response [39]. Although the TTO question required more attention compared to the other two assessments, all participants considered the trade-off between life years and health. Nevertheless, a few participants were unsure of where to put the mark on the line (0–10 years). Some participants found it difficult to imagine or relate to hypothetical health states (in particular the concept of full health) involved in the valuation of current health, which is in line with previous qualitative research on valuations of hypothetical health states [20–22].

Other examples of commonalities with valuations of hypothetical states were that participants relied on both experience and imagination, and took into account personal circumstances and consequences, relationships, ability to support others, and goal achievement [20]. Some of these factors have previously been referred to as non-health factors [20]. Yet, our overall findings from the EQ-5D-5L, EQ VAS and TTO assessments imply that aspects such as social functioning, job satisfaction, expectations of future health, and emotional well-being are incorporated into the concept of one’s health.

Individual responses on the EQ-5D-5L descriptive system, EQ VAS, and TTO did not always follow similar patterns. The differences may be explained by the use of different time perspectives, reference points and comparators, as well as the different health aspects that were considered in the assessments. In the EQ VAS assessment, participants included all important health aspects that they perceived were not covered by the EQ-5D-5L descriptive system, such as stress, well-being, relationships, job satisfaction, and fitness. The TTO assessment differed from the EQ-5D-5L and EQ VAS assessments by more clearly integrating the impact on others, goal achievement, and expectations on future health. Participants thought of how to maximize the time spent with family and friends, and considered the consequences for others. Similar results were found in a previous study of hypothetical health states where respondents more noticeably considered the impact on family members in the TTO method than in the VAS method [23]. Based on previous research, factors and respondent characteristics associated with the willingness to trade off life years include sex, age, subjective life expectancy, preference for quantity over quality of life, living with someone, and having children [42–44]. In addition, the influence of cultural aspects and beliefs has been discussed in previous literature [45].

### Strengths and limitations

The think aloud technique relies on the interviewee’s ability to articulate their own thoughts [29]. A strength of this methodology is the interaction between the participant and the interviewer that may have encouraged thought processes that might not have been present if the participant were to respond to a survey independently. Participants in this study were generally able to think aloud (e.g. by giving examples and describing their experiences) and completed all assessments, with one exception where an interview was stopped by the interviewer after the EQ VAS assessment since the participant seemed uncomfortable with thinking aloud.

Yet, there are limitations to consider. As the assessments are applied in various populations, the inclusion criteria were purposefully broad. The study sample was relatively diverse regarding most of the background characteristics, although with the key exception that all participants had completed a moderate to high level of education. The broad inclusion criterion for age (18‒70 years) was one of the main reasons for conducting additional sampling. However, the upper age limit was not followed in one of the interviews. The interview was included in the analysis since there was no theoretical justification behind the exact upper age limit.

Although the purposeful sampling included patients with a chronic disease that might impact several health dimensions and everyday life, most participants reported no or mild problems on the EQ-5D-5L descriptive system. At the same time, 13 participants stated that they currently had other health problems. Since no other health measure was available, it is uncertain whether the participants in fact were in good health, whether they had adapted their everyday life, or whether they experienced health problems that were not captured by the instrument. Health status of the participants was not included in the assessment of information power. Further research is needed to examine whether respondents’ thought processes differ depending on the severity level of the currently experienced health state or depending on the expected progression of the disease.

Attention should also be paid to the sequence of the assessments due to the possible influence of using introductory questions regarding diabetes, and to the fact that the ability to think aloud may have improved or worsened during the interview. Participants typically compared the three assessments towards the end of the interview, which was handled by conducting a common analysis.

Finally, the transferability of results is an important consideration in qualitative research, as it concerns to what extent the findings of the study can be applied beyond the specific context it was conducted in [46]. The study findings can mainly be applicable for populations with chronic conditions controlled through treatment and self-management, and in contexts with similar health care systems. Thus, there is a need for replication studies in other patient groups and populations. In addition, the results addressing the EQ VAS and TTO assessments are primarily transferable for application of similar design, for example in terms of labelling of end points in both assessments, and for open-ended TTO procedure.

## Conclusions

This study provides insights as to how responses to the EQ-5D-5L, EQ VAS, and TTO are complementary and where these assessments differ in adults with a chronic condition. Participants actively engaged in the assessments, yet found it challenging to translate the wider view of their own health status into a standardized format. The desire to explain the reasoning behind their answers and to discuss potential improvements was expressed. Furthermore, different recall periods and time perspectives were applied in the assessments, independently of the instructions. Thoughts about future personal goal achievement and consequences for others were more clearly integrated in the TTO assessment. The concepts of the best imaginable health state and full health were found difficult to imagine, define, or relate to. Thus, several findings are in line with previous qualitative research on valuations of hypothetical health states. The study findings may contribute to a better understanding of the quantitative results from using EQ-5D-5L, EQ VAS, and TTO to report and value one’s own health, and contribute to the literature regarding possible explanations for differences in health state values depending on the valuation method.

## Data Availability

The data generated and analyzed for the current study are not publicly available. Due to the sensitive nature of the questions asked in this study, participants were assured raw data would remain confidential and would not be shared.

## Abbreviations

PROMs: Patient-reported outcome measures
TTO: Time trade-off
VAS: Visual analogue Scale

## References

[1] Appleby J, Devlin NJ, Parkin DW (2016). Using patient reported outcomes to improve health care.

[2] Snyder CF, Aaronson NK, Choucair AK, Elliott TE, Greenhalgh J, Halyard MY (2012). Implementing patient-reported outcomes assessment in clinical practice: a review of the options and considerations. Qual Life Res.

[3] Drummond M (2015). Methods for the economic evaluation of health care programmes.

[4] Rabin R, de Charro F (2001). EQ-5D: a measure of health status from the EuroQol Group. Ann Med.

[5] Torrance GW, Thomas WH, Sackett DL (1972). A utility maximization model for evaluation of health care programs. Health Serv Res.

[6] Attema AE, Brouwer WBF (2013). In search of a preferred preference elicitation method: a test of the internal consistency of choice and matching tasks. J Econ Psychol.

[7] Arnesen T, Trommald M (2005). Are QALYs based on time trade-off comparable?—A systematic review of TTO methodologies. Health Econ.

[8] Versteegh MM, Brouwer WBF (2016). Patient and general public preferences for health states: a call to reconsider current guidelines. Soc Sci Med.

[9] Brazier J, Rowen D, Karimi M, Peasgood T, Tsuchiya A, Ratcliffe J (2018). Experience-based utility and own health state valuation for a health state classification system: why and how to do it. Eur J Health Econ.

[10] Cubi-Molla P, Shah K, Burstrom K (2018). Experience-based values: a framework for classifying different types of experience in health valuation research. Patient.

[11] Dolan P (2008). Developing methods that really do value the 'Q' in the QALY. Health Econ Policy Law.

[12] Helgesson G, Ernstsson O, Åström M, Burström K (2020). Whom should we ask? A systematic literature review of the arguments regarding the most accurate source of information for valuation of health states. Qual Life Res.

[13] Whalley D, Globe G, Crawford R, Doward L, Tafesse E, Brazier J (2018). Is the EQ-5D fit for purpose in asthma? Acceptability and content validity from the patient perspective. Health Qual Life Outcomes.

[14] Bailey C, Kinghorn P, Orlando R, Armour K, Perry R, Jones L (2016). "The ICECAP-SCM tells you more about what I'm going through': a think-aloud study measuring quality of life among patients receiving supportive and palliative care. Palliative Med.

[15] van Leeuwen KM, Jansen APD, Muntinga ME, Bosmans JE, Westerman MJ, van Tulder MW (2015). Exploration of the content validity and feasibility of the EQ-5D-3L, ICECAP-O and ASCOT in older adults. BMC Health Serv Res.

[16] Matza LS, Boye KS, Stewart KD, Curtis BH, Reaney M, Landrian AS (2015). A qualitative examination of the content validity of the EQ-5D-5L in patients with type 2 diabetes. Health Qual Life Outcomes.

[17] Krig S, Astrom M, Kulane A, Burstrom K (2020). Acceptability of the health-related quality of life instrument EQ-5D-Y-5L among patients in child and adolescent psychiatric inpatient care. Acta Paediatr.

[18] Shah KK, Mulhern B, Longworth L, Janssen MF (2017). Views of the UK general public on important aspects of health not captured by EQ-5D. Patient.

[19] Devlin NJ, Hansen P, Selai C (2004). Understanding health state valuations: a qualitative analysis of respondents' comments. Qual Life Res.

[20] Karimi M, Brazier J, Paisley S (2017). How do individuals value health states? A qualitative investigation. Soc Sci Med.

[21] Mulhern B, Bansback N, Brazier J, Buckingham K, Cairns J, Devlin N, et al. Preparatory study for the revaluation of the EQ-5D tariff: methodology report. Health Technol Assess. 2014;18(12):vii–xxvi, 1–191. 10.3310/hta18120 PMC4781204 24568945

[22] Papageorgiou K, Vermeulen KM, Leijten FRM, Buskens E, Ranchor AV, Schroevers MJ (2015). Valuation of depression co-occurring with a somatic condition: feasibility of the time trade-off task. Health Expect.

[23] Robinson A, Dolan P, Williams A (1997). Valuing health status using VAS and TTO: What lies behind the numbers?. Soc Sci Med.

[24] Greenhalgh J, Dalkin S, Gibbons E, Wright J, Valderas JM, Meads D (2018). How do aggregated patient-reported outcome measures data stimulate health care improvement? A realist synthesis. J Health Serv Res Po.

[25] Anell A, Glenngard AH, Merkur S (2012). Sweden health system review. Health Syst Transit.

[26] Population Statistics: Statistics Sweden; 2019. https://www.scb.se/en/finding-statistics/statistics-by-subject-area/population/population-composition/population-statistics/.

[27] Tong A, Sainsbury P, Craig J (2007). Consolidated criteria for reporting qualitative research (COREQ): a 32-item checklist for interviews and focus groups. Int J Qual Health Care.

[28] Sandelowski M (2000). Whatever happened to qualitative description?. Res Nurs Health.

[29] Drennan J (2003). Cognitive interviewing: verbal data in the design and pretesting of questionnaires. J Adv Nurs.

[30] Herdman M, Gudex C, Lloyd A, Janssen MF, Kind P, Parkin D (2011). Development and preliminary testing of the new five-level version of EQ-5D (EQ-5D-5L). Qual Life Res.

[31] Burstrom K, Sun S, Gerdtham UG, Henriksson M, Johannesson M, Levin LA (2014). Swedish experience-based value sets for EQ-5D health states. Qual Life Res.

[32] Burstrom K, Teni FS, Gerdtham UG, Leidl R, Helgesson G, Rolfson O (2020). Experience-based Swedish TTO and VAS value sets for EQ-5D-5L health states. Pharmacoeconomics.

[33] Burstrom K, Johannesson M, Diderichsen F (2006). A comparison of individual and social time trade-off values for health states in the general population. Health Policy.

[34] Lundberg L, Johannesson M, Isacson DGL, Borgquist L (1999). Health-state utilities in a general population in relation to age, gender and socioeconomic factors. Eur J Public Health.

[35] Dolan P (2011). Thinking about it: thoughts about health and valuing QALYs. Health Econ.

[36] Malterud K, Siersma VD, Guassora AD (2016). Sample size in qualitative interview studies: guided by information power. Qual Health Res.

[37] Braun V, Clarke V (2006). Using thematic analysis in psychology. Qual Res Psychol.

[38] WHO. Constitution of the World Health Organization 1948 [cited 2019 July 9]. https://www.who.int/about/who-we-are/constitution.

[39] Rapkin BD, Schwartz CE (2004). Toward a theoretical model of quality-of-life appraisal: Implications of findings from studies of response shift. Health Qual Life Outcomes.

[40] Schwartz CE, Andresen EM, Nosek MA, Krahn GL (2007). Measurement REPoHS. Response shift theory: important implications for measuring quality of life in people with disability. Arch Phys Med Rehabil.

[41] Heintz E, Wirehn AB, Peebo BB, Rosenqvist U, Levin LA (2012). QALY weights for diabetic retinopathy—a comparison of health state valuations with HUI-3, EQ-5D, EQ-VAS, and TTO. Value Health.

[42] van Nooten FE, van Exel NJ, Koolman X, Brouwer WB (2015). "Married with children" the influence of significant others in TTO exercises. Health Qual Life Outcomes.

[43] van Nooten FE, Houghton K, van Exel J, van Agthoven M, Brouwer WBF, Stull DE (2017). A (latent) class of their own: response patterns in trading off quantity and quality of life in time trade-off exercises. Value Health.

[44] Heintz E, Krol M, Levin LA (2013). The impact of patients' subjective life expectancy on time tradeoff valuations. Med Decis Mak.

[45] Zhuo L, Xu L, Ye J, Sun S, Zhang Y, Burstrom K (2018). Time trade-off value set for EQ-5D-3L based on a Nationally Representative Chinese Population Survey. Value Health.

[46] Malterud K (2001). Qualitative research: standards, challenges, and guidelines. Lancet.

